# Leptin deficiency in maltreated children

**DOI:** 10.1038/tp.2014.79

**Published:** 2014-09-23

**Authors:** A Danese, R Dove, D W Belsky, J Henchy, B Williams, A Ambler, L Arseneault

**Affiliations:** 1 MRC Social, Genetic, and Developmental Psychiatry (SGDP) Centre, Institute of Psychiatry, King's College London, London, UK; 2 Department of Child and Adolescent Psychiatry, Institute of Psychiatry, King's College London, London, UK; 3 National & Specialist Child Traumatic Stress & Anxiety Clinic, South London and Maudsley NHS Foundation Trust, London, UK; 4 Center for the Study of Aging and Human Development, Duke University Medical Center, Durham, NC, USA; 5 Department of Psychology and Neuroscience, Duke University, Durham, NC, USA; 6 Department of Psychiatry and Behavioral Sciences, Duke University, Durham, NC, USA; 7 Institute for Genome Sciences and Policy, Duke University, Durham, NC, USA

## Abstract

Consistent with findings from experimental research in nonhuman primates exposed to early-life stress, children exposed to maltreatment are at high risk of detrimental physical health conditions, such as obesity and systemic inflammation. Because leptin is a key molecule involved in the regulation of both energy balance and immunity, we investigated abnormalities in leptin physiology among maltreated children. We measured leptin, body mass index and C-reactive protein in 170 12-year-old children members of the Environmental-Risk Longitudinal Twin Study, for whom we had prospectively-collected information on maltreatment exposure. We found that maltreated children exhibited blunted elevation in leptin levels in relation to increasing levels of physiological stimuli, adiposity and inflammation, compared with a group of non-maltreated children matched for gender, zygosity and socioeconomic status. These findings were also independent of key potential artifacts and confounders, such as time of day at sample collection, history of food insecurity, pubertal maturation and depressive symptoms. Furthermore, using birth weight as a proxy measure for leptin, we found that physiological abnormalities were presumably not present at birth in children who went on to be maltreated but only emerged over the course of childhood, after maltreatment exposure. Leptin deficiency may contribute to onset, persistence and progression of physical health problems in maltreated children.

## Introduction

Maltreated children experience detrimental physical health consequences, such as obesity and systemic inflammation,^1,2^ consistent with findings from experimental research in nonhuman primates exposed to early-life stress.^3,4^ Because leptin is a key molecule involved in the regulation of both energy balance and immunity, we investigated possible differences in leptin physiology in maltreated vs non-maltreated children.

Leptin is secreted by adipocytes in response to increasing levels of adiposity.^5^ Leptin secretion stimulates hypothalamic neurons inducing downregulation of the neuropeptide Y and upregulation of the alpha-melanocyte-stimulating hormone, inhibits mesolimbic dopamine release and activates the sympathetic nervous system. Through this neuroendocrine response, leptin secretion promotes decrease in energy intake and increase in energy expenditure, ultimately reducing its original secretory stimulus, adiposity. This negative feedback mechanism is disrupted in mice lacking the leptin gene^5^ (the ob/ob mice), who have deficient leptin response to increasing levels of adiposity and, thus, persist in overeating despite becoming obese.

Leptin is also secreted by adipocytes and immune cells in response to increasing levels of systemic inflammation (for example, during infections).^6^ The effects of leptin on immunity are complex and less well understood. Leptin secretion appears to potentiate the activity of both innate immune cells (neutrophils, monocytes/macrophages, natural killer cells) and adaptive immune cells (T-helper 1 cells). By potentiating the immune response and modulating cytokine secretion, leptin may promote effective resolution of the acute inflammatory response, ultimately reducing its original secretory stimulus. This potential negative feedback mechanism also appears to be disrupted in ob/ob mice,^6^ who have deficient leptin response to increasing levels of inflammation and, thus, show more severe outcomes after administration of pro-inflammatory stimuli (for example, lipopolysaccharide, interleukin-1b, tumor necrosis factor-alpha, ozone).

Variation in leptin physiology could be induced not only by genetic influences, like in the case of ob/ob mice or humans with congenital leptin deficiency,^7,8^ but also by experiences during early development.^9^ For example, mice exposed to early-life stress showed later blunted leptin response to homologous stimulation with psychological stressors, such as chronic forced swimming or chronic restrain.^10^ In the present study we provide initial evidence that the effects of early-life stress on leptin reactivity described in experimental animal models translate to humans, and that effect modification by early-life stress also applies to later heterologous physiological stimulation by adiposity and inflammation, consistent with abnormalities described in ob/ob mice.

## Materials and Methods

### Sample

Participants were members of the Environmental-Risk Longitudinal Twin Study, which tracks the development of a nationally representative birth cohort of 2232 British children born during 1994–1995.^11^ Families were recruited to represent the UK population of families with newborns in the 1990s, on the basis of (a) residential location throughout England and Wales and (b) mother's age (that is, older mothers having twins via assisted reproduction were under-selected and teenaged mothers with twins were over-selected). We used this sampling (a) to replace high-risk families who were selectively lost to the register via nonresponse and (b) to ensure sufficient numbers of children growing up in high-risk environments. Follow-up home visits were conducted when the children were aged 7 years (98% participation), 10 years (96%) and 12 years (96%).

At age 12 years, we collected blood samples from children living in 41 homes where we had previously found evidence of physical maltreatment. We also collected blood samples from children living in 46 homes where maltreatment did not take place.^12^ These maltreatment-free homes were selected from the cohort to match the homes with maltreatment based on family socioeconomic status, gender and zygosity. The validity of our matching procedure was supported by the lack of differences in matching variables between children from homes with (*n*=82) and without (*n*=92) maltreatment. The sampling for the current study was restricted to children with no medical conditions that could have influenced inflammation biomarker levels at the time of assessment (for example, asthma, cold). One hundred and seventy-two dried blood spot specimens (from 81 maltreated and 91 non-maltreated children) were available for leptin and CRP analysis.

The study protocol was approved by the Joint South London and Maudsley and the Institute of Psychiatry Research Ethics Committee. Parents gave informed consent and children gave assent to participate in the study.

### Maltreatment assessment

Methods used to assess childhood maltreatment in our sample have been described in detail elsewhere.^13^ We assessed physical maltreatment by an adult^14^ using a standardized clinical interview protocol designed to enhance mothers' comfort with reporting valid child maltreatment information, while also meeting researchers' responsibilities for referral under the UK Children Act. No family has left the study after intervention. When mothers reported any maltreatment, interviewers followed with standardized probes (for example, accidental harm was ruled out; harm by age peers was coded as bullying, not maltreatment). Sexual abuse was queried directly. Over the years of data collection, the study maintained a cumulative dossier for each child, composed of recorded debriefings with interviewers who had coded any indication of maltreatment at any of the four successive home visits, recorded narratives of the four successive caregiver interviews at child ages 5, 7, 10 and 12 years (covering the period from birth to 12 years), and information from clinicians whenever the study made a referral. On the basis of the review of each child's cumulative dossier, two clinical psychologists (Professor Terrie E Moffitt and the project coordinator) reached consensus for whether physical maltreatment had occurred. Examples of maltreatment in environmental-risk children included the following: the mother smacked the child weekly, leaving marks or bruises; child was repeatedly beaten by a young adult stepsibling; child was routinely smacked by father when drunk, ‘just to humiliate him' child was fondled sexually and often slapped by the mothers' boyfriend. Many, but not all, cases identified in the course of our research were under investigation by the police or social services, already on the child-protection register, or in foster care at follow-up, having been taken away from their parents because of abuse.

### Biomarker assessment

#### Sample collection

Biomarkers were assessed in dried blood spots collected during a home visit at age 12.^12^ Children's fingers were sterilized with an alcohol swab and incised with a Tenderlett Finger incision device (Elitech, Berkhamsted, UK). The first blood drop was discarded on tissue. Five subsequent drops of 50 μl each were collected on a Whatman 903 Protein Saver Card (VWR, Lutterworth, UK). Cards were immediately transferred to an air-tight drying box containing two Maxipax indicating absorbent packets each containing 10 g silica gel. Samples were allowed to dry completely for 5 h before being transferred into small Ziploc bags and stored at room temperature. Within 14 days of collection, samples were transported to the laboratory for storage at −20 ºC. For analysis, dried blood spots were eluted into buffer solution and biomarkers levels were measured with commercially available ELISA kit.

Leptin levels (pg ml^−1^) were measured in dried blood spots^15^ using a high-sensitivity ELISA kit (Invitrogen, Paisley, UK, Cat# KAC2281) following the protocol recommended by the manufacturer. Samples were analyzed in duplicate, giving a mean coefficient of variation of 4.42%. Manufacturer's intra- and inter-assay variation were 3.9 and 5.3%, respectively.

C-reactive protein levels (mg l^−1^) were measured in dried blood spots^16^ using a high-sensitivity ELISA kit (Quantikline ELISA Kit DCRP00, R&D Systems, Minneapolis, MN, USA) following the protocol recommended by the manufacturer. Samples were analyzed in duplicate, giving a mean coefficient of variation of 5.2%. Manufacturer's intra- and inter-assay variation were 5.5 and 6.5%, respectively.

### Other study variables

Height and weight were measured by trained interviewers at age 12 years during home visits. Body mass index (BMI) was then computed as weight in kilograms over squared height in meters.

Birth weight was extracted from birth records. Because birth weight is significantly and positively associated with leptin levels at birth,^17^ we used birth weight as a proxy measure for leptin levels at birth.

The family socioeconomic status at the age of 5 years was defined through a standardized composite of parental income, education and occupation. The three socioeconomic status indicators were highly correlated (*r*=0.57–0.67) and loaded significantly onto one latent factor. The population-wide distribution of the resulting factor was divided in tertiles for analyses.

Zygosity was determined using a standard zygosity questionnaire, which has been shown to have 95% accuracy.^18^ Ambiguous cases were zygosity-typed using DNA.

Time of the day at blood spot collection (in minutes) was imputed from the home visits' records.

History of food insecurity was reported by the mother to a clinical interviewer when children were aged 7–10 years using a seven-item scale developed by the US Department of Agriculture.^19^ Using both assessments available to us, we identified families that were ‘ever food insecure' (food insecure at age 7 and/or age 10 years assessments) and compared them with those that were always food secure.

Pubertal maturation at age 12 years was evaluated through maternal ratings of Tanner's stages during home visits. Sex-specific variables were combined to obtain an overall index of pubertal maturation for each study member.

Depressive symptoms at age 12 years were assessed using the Children's Depression Inventory.^20^ This self-report scale has been widely used in both clinical and research settings to measure cognitive, affective and behavioral signs of depression in school-age children and adolescents (age 7–17 years).

### Statistical analysis

Because the sample-wide distribution of leptin and CRP levels were positively skewed, they were log-transformed to approximate normality.

The groups of maltreated and non-maltreated children were frequency-matched for family socioeconomic status, gender and zygosity. All analyses were then adjusted for these matching variables. Significance testing for group differences was performed using ordinary least-square regression analyses adjusted for the effect of familial clustering to correct for the inclusion of two study children in each family (using the command regress and option cluster in STATA SE, 12th edition).

To test whether maltreatment exposure could significantly modify leptin response to key secretagogue stimuli, adiposity and inflammation, we tested whether the strength of the association between leptin and BMI or CRP was different in maltreated compared to non-maltreated children (that is, the significance of the interaction term).

To test whether results were dependent on distribution assumption (that is, normality), we repeated the analyses using nonparametric analyses by quantile regression with bootstrapped confidence intervals to account for familial clustering (using the command qreg in STATA SE, 12th edition).

To test whether the results were accounted for by key potential intervening variables, we repeated the regression analyses adding in turn key variables (that is, time of the day at assessment, history of food insecurity, pubertal maturation or depressive symptoms) and two additional interaction terms into a fully saturated model.

## Results

**Figure 1:**
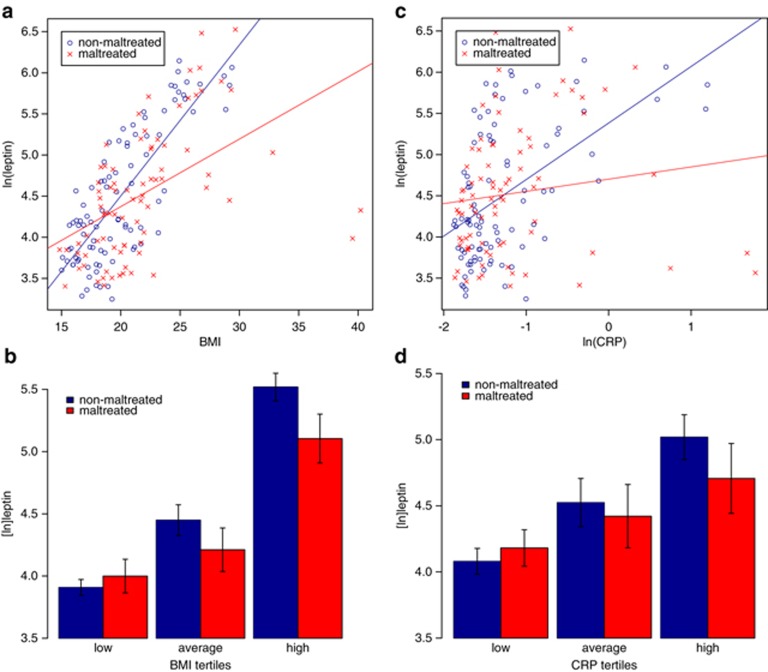
Blunted leptin response to increasing BMI and CRP levels in maltreated children compared with matched non-maltreated children. (**a**) Association between a continuous BMI measure and leptin levels in the two groups (interaction beta=−1.29, *P*=0.002). (**b**) Association between increasing tertiles of the sample-wide BMI distribution and leptin levels in the two groups (interaction *P*=0.012). (**c**) Association between a continuous CRP measure and leptin levels in the two groups (interaction beta=−0.50, *P*<0.001). (**d**) Association between increasing tertiles of the sample-wide CRP distribution and leptin levels in the two groups (interaction *P*<0.001). BMI, body mass index; CRP, C-reactive protein.

We observed the expected significant positive association between BMI and leptin (beta=0.52, *P*<0.001). Although we found no main effect of maltreatment on leptin levels (BMI-adjusted: beta=−0.08, *P*=0.143), the association between BMI and leptin was modified by maltreatment (interaction beta=−1.29, *P*=0.002; see Figure 1a), with maltreated children (beta=0.41, *P*=0.007) showing blunted elevation in leptin levels in relation to increasing BMI levels compared with non-maltreated children (beta=0.72, *P*<0.001). Nonparametric analysis with quantile regression revealed that effect modification was not simply due to potential deviations from normal distribution (interaction *P*=0.012; see Figure 1b).

We also observed the expected significant positive association between inflammation and leptin (beta=0.30, *P*=0.002). This association was also modified by maltreatment status (interaction beta=−0.50, *P*<0.001; see Figure 1c), with maltreated children (beta=0.10, *P*=0.281) showing blunted elevation in leptin levels in relation to increasing CRP levels compared with non-maltreated children (beta=0.49, *P*<0.001). Nonparametric analysis with quantile regression showed that effect modification was not simply due to potential deviations from normal distribution (interaction *P*<0.001; see Figure 1d).

The blunted leptin response to increasing levels of adiposity or inflammation in maltreated vs non-maltreated children was not influenced by key potential artifacts and confounders, such as the time of day at sample collection, history of food insecurity, pubertal maturation or depressive symptoms.

First, it has been shown that leptin secretion has circadian variation,^21^ and, thus, variation in time of the day at blood collection might have biased the observed results. However, even when statistically controlling for time of the day at collection, maltreated children showed blunted leptin responses to increasing adiposity (interaction beta=−1.37, *P*=0.001) and inflammation (interaction beta=−0.57, *P*<0.001).

Second, because adverse family environment has been associated with food insecurity^19^ and nutritional signals during development may influence leptin levels,^9^ a history of food insecurity might have confounded the effects of maltreatment on leptin physiology. We found that maltreated children were more likely to have experienced food insecurity than non- maltreated children (odds ratio=4.20; 95% confidence interval=1.43–12.4, *P*=0.009), but food insecurity was unrelated to leptin levels (beta=−0.06, *P*=0.502). Even when statistically controlling for experiences of food insecurity, maltreated children showed blunted leptin responses to increasing adiposity (interaction beta=−0.99, *P*=0.003) and inflammation (interaction beta=−0.52, *P*<0.001).

Third, because childhood maltreatment has been associated with earlier pubertal maturation^22^ and pubertal maturation has been associated with variation in leptin levels,^23^ pubertal maturation might have confounded the effects of maltreatment on leptin physiology. We found that maltreated children showed a nonsignificant advancement in pubertal maturation compared with non-maltreated children (beta=0.15, *P*=0.164), and more advanced pubertal maturation was linked to higher leptin levels (beta=0.19, *P*=0.025). Even when statistically controlling for pubertal maturation, maltreated children showed blunted leptin responses to increasing adiposity (interaction beta=−1.25, *P*=0.001) and inflammation (interaction beta=−0.32, *P*=0.054).

Finally, because childhood maltreatment has been associated with depression risk^24^ and depressive symptoms have been associated with variation in leptin levels in some studies of adults,^25^ depressive symptoms might have confounded the effects of maltreatment on leptin physiology. Maltreated children showed a nonsignificant elevation in depressive symptoms compared to non-maltreated children (beta=0.15, *P*=0.107), and depressive symptoms were not associated with leptin levels in children (beta=−0.04, *P*=0.468). Even when statistically controlling for depressive symptoms, maltreated children showed blunted leptin responses to increasing adiposity (interaction beta=−1.29, *P*=0.003) and inflammation (interaction beta=−0.38, *P*=0.026).

We also considered that group differences in leptin secretion could have preceded maltreatment exposure. If that was the case, the findings reported here could have been simply explained by preexisting and stable differences in leptin physiology rather than by maltreatment exposure. We were unable to assess leptin physiology before maltreatment exposure, at birth. However, birth weight is significantly and positively correlated with leptin levels at birth (*r*=0.46, 95% confidence interval=0.43–0.50).^17^ We therefore used birth weight as a proxy measure for leptin levels at birth, before maltreatment exposure.

We found that maltreated and non-maltreated children did not show significant differences in birth weight (beta=−0.10, *P*=0.338). To the extent that birth weight is a valid proxy measure, these findings suggest that differences in leptin secretion were not present in children before maltreatment exposure.

Furthermore, if maltreatment exposure was linked to subsequent impairment in leptin response to physiological stimuli, maltreated children would have shown greater intra-individual changes in body weight than non-maltreated children. Consistent with the adipogenic effect of leptin deficiency, we found that maltreated children showed larger weight gain than non-maltreated children between birth and age 12 years (beta=0.21, *P*=0.030), suggesting that leptin deficiency may have emerged in maltreated children after maltreatment exposure.

## Discussion

We found that maltreated children exhibited blunted elevation in leptin levels in relation to increasing levels of two key physiological stimuli, namely adiposity and inflammation. Leptin deficiency in maltreated children was not explained by important potential artifacts and confounders, and presumably emerged only after maltreatment exposure. These results are consistent with findings of blunted leptin response reported in experimental research in animals exposed to early-life stress.^10^ Although a study of rats suggested that early-life stress might also be linked to lower baseline leptin levels,^26^ this was not observed in a previous study in nonhuman primates^27^ and in our study of children. Furthermore, these results are consistent with previous evidence of insufficient glucocorticoid signaling in animals and humans exposed to early-life stress,^28, 29, 30^ which could lead to reduced stimulatory effects of glucocorticoids on leptin secretion.^31,32^ These results have implications for understanding pathophysiology and preventing disease in maltreated individuals.

Results suggest putative biological mechanisms through which exposure to child maltreatment could be translated into risk for later obesity.^1^ First, blunted leptin response in maltreated children could lessen leptin-dependent inhibition of appetite. As in ob/ob mice,^5^ leptin deficiency could lead maltreated children to overeat despite becoming obese. Second, blunted leptin response in maltreated children may impair the development of hypothalamic neurons regulating feeding. Research showed that ob/ob mice have a permanent disruption of neural projection pathways from the arcuate nucleus of the hypothalamus, which could be reversed by administration of exogenous leptin in early life but not adult life.^33^ Consistent with these experimental findings, leptin deficiency during sensitive developmental windows could have enduring consequences on feeding regulation in maltreated children. Similar mechanisms might also help explain the effects of child maltreatment on inflammation,^2^ but the links between leptin and inflammation are less well understood and require further investigation.

Results also offer new insights on potential strategies to remediate the detrimental heath consequences of child maltreatment. On one hand, chronic leptin replacement normalizes metabolic and immune abnormalities in ob/ob mice.^5,6^ However, chronic administration of exogenous leptin could also contribute to leptin resistance by downregulating cellular response to leptin^34^ and, thus, could potentially worsen related health outcomes. On the other hand, if the core difference in maltreated children is a reduced trophic function of leptin during brain development, it is possible that timely, short-term leptin replacement in childhood could prevent later health consequences. Clearly, more basic and translational research is needed before clinical application of these findings. Future research should also test whether early psychosocial interventions targeting recurrence of maltreatment and its related psychological impairments^35^ can remediate differences in leptin secretion.

Early identification and remediation of biological abnormalities in maltreated children may help prevent later disease.^28,36,37^ Although leptin deficiency is already detectable in maltreated children, clinical outcomes, such as obesity, may only become apparent in later life.^1^ The 'incubation' period between exposure and clinical outcomes could offer clinicians a window of opportunity to lessen biological vulnerabilities like leptin deficiency in maltreated children, and, thus, to prevent later disease. The initial findings reported here call for further research to understand the possible contribution of leptin to health problems in maltreated children.
